# *catena*-Poly[sodium(I)-μ-tetra­butoxy­borato]

**DOI:** 10.1107/S1600536809012100

**Published:** 2009-04-08

**Authors:** Graeme J. Gainsford, Tim Kemmitt

**Affiliations:** a Industrial Research Limited, PO Box 31-310, Lower Hutt, New Zealand

## Abstract

The title compound, [Na(C_16_H_36_BO_4_)]_*n*_, has a fourfold  axis passing through the Na and B atoms which both are bound by four O atoms. The tetra­butoxy­borate anion provides the bridging to form one-dimensional polymers running along [001], just like those found for the tetra­ethoxy­borate structure. The two but­oxy ‘tail’ atoms are disordered over two conformations in a 0.887 (9):0.113 (9) ratio.

## Related literature

For general background to the potential applications of boron diolates and alkoxides in hydrogen storage/recycling systems, see: Kemmitt & Gainsford (2009 ▶). For related structures, see: Gainsford & Kemmitt (2004 ▶, 2005 ▶); Bishop *et al.* (2000 ▶); Caselli *et al.* (2000 ▶); Zviedre & Belsky (2001 ▶). For a description of the Cambridge Structural Database, see: Allen (2002 ▶).
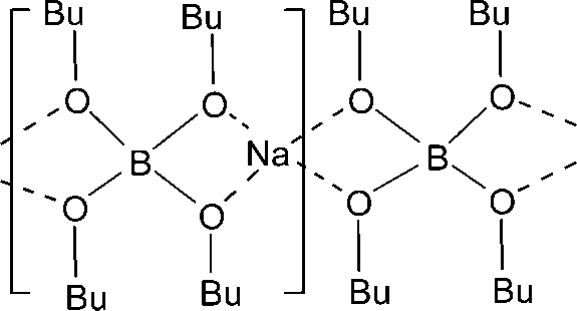


## Experimental

### 

#### Crystal data

[Na(C_16_H_36_BO_4_)]
*M* *_r_* = 326.25
Tetragonal, 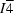

*a* = 13.3552 (17) Å
*c* = 5.7422 (6) Å
*V* = 1024.2 (2) Å^3^
*Z* = 2
Mo *K*α radiation
μ = 0.09 mm^−1^
*T* = 112 K
0.80 × 0.32 × 0.10 mm

#### Data collection

Bruker APEXII CCD diffractometer
Absorption correction: multi-scan (*SADABS*; Sheldrick, 2003 ▶) *T*_min_ = 0.821, *T*_max_ = 0.992
3660 measured reflections
906 independent reflections
724 reflections with *I* > 2σ(*I*)
*R*_int_ = 0.027

#### Refinement

*R*[*F* ^2^ > 2σ(*F* ^2^)] = 0.049
*wR*(*F* ^2^) = 0.145
*S* = 1.05
906 reflections
66 parameters
3 restraints
H-atom parameters constrained
Δρ_max_ = 0.29 e Å^−3^
Δρ_min_ = −0.20 e Å^−3^

### 

Data collection: *APEX2* (Bruker, 2005 ▶); cell refinement: *APEX2* and *SAINT* (Bruker, 2005 ▶); data reduction: *SAINT*; program(s) used to solve structure: *SHELXS97* (Sheldrick, 2008 ▶); program(s) used to refine structure: *SHELXL97* (Sheldrick, 2008 ▶); molecular graphics: *PLATON* (Spek, 2009 ▶); software used to prepare material for publication: *SHELXL97* and *PLATON*.

## Supplementary Material

Crystal structure: contains datablocks global, I. DOI: 10.1107/S1600536809012100/bq2133sup1.cif

Structure factors: contains datablocks I. DOI: 10.1107/S1600536809012100/bq2133Isup2.hkl

Additional supplementary materials:  crystallographic information; 3D view; checkCIF report

## Figures and Tables

**Fig. 1.**
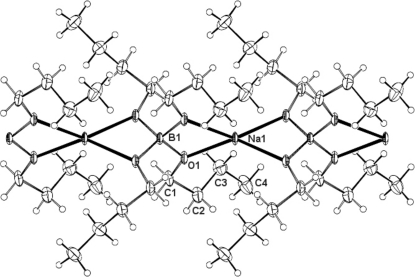
PLATON ORTEP view (Spek, 2009) of the cell contents (30% probability ellipsoids) showing the one-dimensional polymers which run the 4-fold inversion c axis. Only the major conformer C3 & C4 atomic positions are shown for clarity.

## supplementary crystallographic information

### Comment

This study is part of a program aimed at investigating boron diolates and alkoxides with potential applications in hydrogen storage/recycling systems (Kemmitt & Gainsford, 2009). Several other related compounds have been reported [Gainsford & Kemmitt, 2004 (GAKLAG); Gainsford & Kemmitt, 2005 (KASSUT); Bishop *et al.*, 2000 (ABAYUX, ABAZEI)]. There are no reported structures for tetrabutoxyborate salts (Allen, 2002), though there are a large number of tetramethoxyborate salts reported (see Gainsford & Kemmitt, 2005).

The basic polymeric fragment of the title compound, with asymmetric unit formula [Na_0.25_(C_4_H_9_B_0.25_O), has 4-fold inversion crystallographic symmetry with the symmetry (*c*) axis passing through the Na & B atoms. Enantiomeric resolution was not expected from the synthesis and it could not be obtained using anomalous dispersion effects.

The sodium cations are four-coordinate in a highly distorted tetrahedral arrangement with mean Na–O 2.2496 (14)Å and O–Na–O angles of 138.10 (5) and 60.75 (7) compared with 138.18 (8) and 60.62 (12)° in analogous tetraethoxyborato structure KASSUT Gainsford & Kemmitt, 2005 C). This Na—O distance is shorter than those reported when the bound O atoms atoms are not structurally constrained; one example of the latter case is POGDAQ01 (Caselli *et al.* (2000)) where Na–O range from 2.243–2.355Å in a niobium-tetraoxycallix(4)arene compound.

The B—O and C—O bond lengths average to 1.416 (4) and 1.467 (4)Å, and the B—O—C angle mean is 117.8 (2)°, values that fall within normal ranges as reported before in GAKLAG (Gainsford & Kemmitt, 2004). The O—B—O angles are distorted from pure tetrahedral values (101.45 (7) & 113.62 (8)°) somewhat more than those in the bis(1,1,1-trihydroxymethylpropane)borate salt (XOCHOM, Zviedre & Belsky, 2001) of 108.6–109.9°. The borate anions bridge the sodium cations making one-dimensional polymers running along the 4-fold inversion symmetry *c* axis direction (Fig. 1). This packing mode was also observed in *catena*-(µ_3_bis(ethylenedioxy)borato)- sodium(I)) (GAKLAG, Gainsford & Kemmitt, 2004) where the polymers were aligned with the 2_1_ screw axis.

### Experimental

NaBO_2_ (5.00 g, 76 mmol) was refluxed in methanol (150 ml) for around 4 h, running the condensate through a bed of molecular sieves before rejoining the reaction flask to remove water liberated from the reaction. The methanol was removed by distillation before adding toluene (80 ml) and n-butanol (80 ml). The solvent volume was reduced to *ca* 50 ml by distillation, and allowed to cool to room temperature. The colourless product appeared as fine needles, which gradually grew in size over several months in a sealed flask subjected to daily ambient temperature cycles. The needles were filtered under nitrogen, and dried *in vacuo*. Yield 23.5, (95%). ^1^H NMR (d_8_-thf 30°C): δ 1.04, (t, 7.3 Hz, CH_3_ 12H); 1.45, (m, CH_2_ 8H); 1.64, (m, CH_2_ 8H); 3.46, (t, 7.2 Hz, CH_2_ 8H) ^13^C NMR (CDCl_3_ 30°C): δ 14.80, (CH_3_); 20.64, (CH_2_); 36.42, (CH_2_); 61.12, (OCH_2_). ^11^B NMR (d_8_-thf 30°C): δ 2.78 p.p.m..

### Refinement

In the absence of significant anomalous scattering, the values of the Flack parameter were indeterminate. Accordingly, the Friedel-equivalent reflections were merged prior to the final refinements. The butyl chain carbon atoms C3 & C4 were disordered over two conformations in a final ratio of (unprimed:primed) 0.887:0.113 (9). Three restraints were applied: distances C2–C3, C3–C4, C2—C4 were restrained to be the same for both conformers. Atoms C3' & C4' were refined with a common isotropic U.

All H atoms were constrained to their expected geometries (C—H 0.99, 0.98 Å) except for the latter refinement cycles when the atoms on minor conformer atom C4' were fixed, with a common isotropic thermal parameter. The H atoms on C2 & C3 were refined with isotropic parameters while H atoms on C4 and C3' were refined with *U*_iso_ 1.5,1.2 times that of the *U*_eq_ of their carrier atoms.

### Crystal data

**Table:**

[Na(C_16_H_36_BO_4_)]	*D*_x_ = 1.058 Mg m^−^^3^
*M**_r_* = 326.25	Mo *K*α radiation, λ = 0.71073 Å
Tetragonal, *I*4	Cell parameters from 1472 reflections
Hall symbol: I -4	θ = 3.1–31.3°
*a* = 13.3552 (17) Å	µ = 0.09 mm^−^^1^
*c* = 5.7422 (6) Å	*T* = 112 K
*V* = 1024.2 (2) Å^3^	Needle, colourless
*Z* = 2	0.80 × 0.32 × 0.10 mm
*F*(000) = 360	

### Data collection

**Table:**

Bruker APEXII CCD diffractometer	906 independent reflections
Radiation source: fine-focus sealed tube	724 reflections with *I* > 2σ(*I*)
graphite	*R*_int_ = 0.027
Detector resolution: 8.333 pixels mm^-1^	θ_max_ = 31.5°, θ_min_ = 3.1°
φ and ω scans	*h* = −19→18
Absorption correction: multi-scan (*SADABS*; Sheldrick, 2003))	*k* = −19→18
*T*_min_ = 0.821, *T*_max_ = 0.992	*l* = −8→4
3660 measured reflections	

### Refinement

**Table:**

Refinement on *F*^2^	Primary atom site location: structure-invariant direct methods
Least-squares matrix: full	Secondary atom site location: difference Fourier map
*R*[*F*^2^ > 2σ(*F*^2^)] = 0.049	Hydrogen site location: inferred from neighbouring sites
*wR*(*F*^2^) = 0.145	H-atom parameters constrained
*S* = 1.05	*w* = 1/[σ^2^(*F*_o_^2^) + (0.0875*P*)^2^ + 0.2269*P*] where *P* = (*F*_o_^2^ + 2*F*_c_^2^)/3
906 reflections	(Δ/σ)_max_ < 0.001
66 parameters	Δρ_max_ = 0.28 e Å^−^^3^
3 restraints	Δρ_min_ = −0.20 e Å^−^^3^

### Special details

**Table:**

Geometry. All e.s.d.'s (except the e.s.d. in the dihedral angle between two l.s. planes) are estimated using the full covariance matrix. The cell e.s.d.'s are taken into account individually in the estimation of e.s.d.'s in distances, angles and torsion angles; correlations between e.s.d.'s in cell parameters are only used when they are defined by crystal symmetry. An approximate (isotropic) treatment of cell e.s.d.'s is used for estimating e.s.d.'s involving l.s. planes.
Refinement. Refinement of *F*^2^ against ALL reflections. The weighted *R*-factor *wR* and goodness of fit *S* are based on *F*^2^, conventional *R*-factors *R* are based on *F*, with *F* set to zero for negative *F*^2^. The threshold expression of *F*^2^ > σ(*F*^2^) is used only for calculating *R*-factors(gt) *etc*. and is not relevant to the choice of reflections for refinement. *R*-factors based on *F*^2^ are statistically about twice as large as those based on *F*, and *R*- factors based on ALL data will be even larger.

### Fractional atomic coordinates and isotropic or equivalent isotropic displacement parameters (Å^2^)

**Table:**

	*x*	*y*	*z*	*U*_iso_*/*U*_eq_	Occ. (<1)
Na1	0.0000	0.5000	0.7500	0.0311 (4)	
O1	0.07286 (11)	0.45588 (11)	0.4120 (2)	0.0321 (4)	
C1	0.15761 (19)	0.4090 (2)	0.3117 (4)	0.0454 (6)	
H1A	0.1934	0.4573	0.2106	0.086 (13)*	
H1B	0.1359	0.3518	0.2144	0.083 (13)*	
C2	0.2267 (2)	0.3726 (2)	0.5005 (5)	0.0490 (7)	
H2A	0.2829	0.3356	0.4284	0.092 (15)*	
H2B	0.1899	0.3254	0.6022	0.073 (11)*	
C3	0.2693 (2)	0.4582 (3)	0.6508 (7)	0.0527 (10)	0.887 (9)
H3A	0.2989	0.5098	0.5480	0.069 (12)*	0.887 (9)
H3B	0.2141	0.4898	0.7394	0.050 (9)*	0.887 (9)
C4	0.3485 (2)	0.4210 (4)	0.8197 (8)	0.0682 (13)	0.887 (9)
H4A	0.3205	0.3671	0.9155	0.102*	0.887 (9)
H4B	0.3699	0.4762	0.9204	0.102*	0.887 (9)
H4C	0.4062	0.3956	0.7323	0.102*	0.887 (9)
B1	0.0000	0.5000	0.2500	0.0279 (8)	
C3'	0.252 (3)	0.395 (3)	0.748 (4)	0.087 (10)*	0.113 (9)
H3'1	0.2743	0.3337	0.8282	0.105*	0.113 (9)
H3'2	0.1924	0.4218	0.8298	0.105*	0.113 (9)
C4'	0.336 (3)	0.473 (3)	0.750 (8)	0.087 (10)*	0.113 (9)
H4'1	0.3076	0.5348	0.6716	0.13 (11)*	0.113 (9)
H4'2	0.3929	0.4510	0.6891	0.13 (11)*	0.113 (9)
H4'3	0.3431	0.4948	0.9211	0.13 (11)*	0.113 (9)

### Atomic displacement parameters (Å^2^)

**Table:**

	*U*^11^	*U*^22^	*U*^33^	*U*^12^	*U*^13^	*U*^23^
Na1	0.0425 (6)	0.0425 (6)	0.0082 (6)	0.000	0.000	0.000
O1	0.0386 (8)	0.0456 (8)	0.0120 (5)	0.0083 (6)	−0.0005 (5)	−0.0011 (6)
C1	0.0477 (13)	0.0700 (17)	0.0186 (9)	0.0186 (12)	−0.0007 (8)	−0.0077 (9)
C2	0.0465 (13)	0.0701 (16)	0.0304 (11)	0.0187 (11)	−0.0042 (11)	−0.0067 (12)
C3	0.0473 (17)	0.060 (2)	0.0508 (18)	−0.0018 (13)	−0.0073 (14)	−0.0031 (15)
C4	0.0479 (17)	0.101 (3)	0.055 (2)	0.0221 (19)	−0.0186 (17)	−0.029 (2)
B1	0.0365 (12)	0.0365 (12)	0.0106 (14)	0.000	0.000	0.000

### Geometric parameters (Å, °)

**Table:**

Na1—O1^i^	2.2496 (14)	C3—H3B	0.9900
Na1—O1^ii^	2.2496 (14)	C4—H4A	0.9800
Na1—O1	2.2496 (14)	C4—H4B	0.9800
Na1—O1^iii^	2.2496 (14)	C4—H4C	0.9800
O1—C1	1.416 (3)	B1—O1^iv^	1.4696 (14)
O1—B1	1.4696 (14)	B1—O1^v^	1.4696 (14)
C1—C2	1.505 (3)	B1—O1^ii^	1.4696 (14)
C1—H1A	0.9900	B1—Na1^vi^	2.8711 (3)
C1—H1B	0.9900	C3'—C4'	1.531 (19)
C2—C3'	1.49 (2)	C3'—H3'1	0.9900
C2—C3	1.541 (4)	C3'—H3'2	0.9900
C2—H2A	0.9900	C4'—H4'1	1.01 (4)
C2—H2B	0.9900	C4'—H4'2	0.89 (5)
C3—C4	1.519 (5)	C4'—H4'3	1.03 (4)
C3—H3A	0.9900		
			
O1^i^—Na1—O1^ii^	138.10 (5)	C4—C3—C2	111.8 (3)
O1^ii^—Na1—O1	60.75 (7)	C4—C3—H3A	109.3
C1—O1—B1	116.68 (14)	C2—C3—H3A	109.3
C1—O1—Na1	144.35 (13)	C4—C3—H3B	109.3
B1—O1—Na1	98.90 (7)	C2—C3—H3B	109.3
O1—C1—C2	109.89 (19)	H3A—C3—H3B	107.9
O1—C1—H1A	109.7	O1^iv^—B1—O1^v^	101.44 (11)
C2—C1—H1A	109.7	O1^iv^—B1—O1^ii^	113.63 (6)
O1—C1—H1B	109.7	C2—C3'—C4'	108 (2)
C2—C1—H1B	109.7	C2—C3'—H3'1	110.1
H1A—C1—H1B	108.2	C4'—C3'—H3'1	110.1
C3'—C2—C1	139.4 (17)	C2—C3'—H3'2	110.1
C1—C2—C3	112.9 (2)	C4'—C3'—H3'2	110.1
C3'—C2—H2A	109.2	H3'1—C3'—H3'2	108.4
C1—C2—H2A	109.0	C3'—C4'—H4'1	106 (3)
C3—C2—H2A	109.0	C3'—C4'—H4'2	113 (3)
C3'—C2—H2B	71.4	H4'1—C4'—H4'2	115 (5)
C1—C2—H2B	109.0	C3'—C4'—H4'3	105 (3)
C3—C2—H2B	109.0	H4'1—C4'—H4'3	104 (3)
H2A—C2—H2B	107.8	H4'2—C4'—H4'3	113 (4)
			
O1^i^—Na1—O1—C1	45.7 (3)	C1—C2—C3—C4	−172.9 (3)
O1^ii^—Na1—O1—C1	176.4 (3)	C1—O1—B1—O1^iv^	60.0 (2)
O1^iii^—Na1—O1—C1	−52.8 (3)	Na1—O1—B1—O1^iv^	−122.34 (3)
O1^i^—Na1—O1—B1	−130.79 (7)	C1—O1—B1—O1^v^	−55.3 (2)
O1^ii^—Na1—O1—B1	0.0	C1—O1—B1—O1^ii^	−177.7 (2)
B1—O1—C1—C2	177.19 (19)	Na1—O1—B1—O1^ii^	0.0
Na1—O1—C1—C2	1.1 (4)	C1—O1—B1—Na1^vi^	2.3 (2)
O1—C1—C2—C3'	−25.0 (19)	Na1—O1—B1—Na1^vi^	180.0
O1—C1—C2—C3	−63.0 (3)	C1—O1—B1—Na1	−177.7 (2)

Symmetry codes: (i) −*y*+1/2, *x*+1/2, −*z*+3/2; (ii) −*x*, −*y*+1, *z*; (iii) *y*−1/2, −*x*+1/2, −*z*+3/2; (iv) *y*−1/2, −*x*+1/2, −*z*+1/2; (v) −*y*+1/2, *x*+1/2, −*z*+1/2; (vi) *x*, *y*, *z*−1.

## References

[bb1] Allen, F. H. (2002). *Acta Cryst.* B**58**, 380–388. 10.1107/s0108768102003890 12037359

[bb2] Bishop, M., Bott, S. G. & Barron, A. R. (2000). *J. Chem. Soc. Dalton Trans.* pp. 3100–3105.

[bb4] Bruker (2005). *APEX2*, *SAINT* and *SADABS* Bruker AXS Inc., Madison, Wisconsin, USA.

[bb5] Caselli, A., Solari, E., Scopelliti, R., Floriana, C., Re, N., Rizoli, C. & Chiesi-Villa, A. (2000). *J. Am. Chem. Soc.* **122**, 3652–3670.

[bb7] Gainsford, G. J. & Kemmitt, T. (2004). *Acta Cryst.* E**60**, m1943–m1944.

[bb8] Gainsford, G. J. & Kemmitt, T. (2005). *Acta Cryst.* C**61**, m417–m418. 10.1107/S0108270105024121 16143763

[bb9] Kemmitt, T. & Gainsford, G. J. (2009). *Int. J. Hydrogen Energ* Submitted.

[bb10] Sheldrick, G. M. (2003). *SADABS* University of Göttingen, Germany.

[bb11] Sheldrick, G. M. (2008). *Acta Cryst.* A**64**, 112–122. 10.1107/S0108767307043930 18156677

[bb12] Spek, A. L. (2009). *Acta Cryst.* D**65**, 148–155. 10.1107/S090744490804362X PMC2631630 19171970

[bb13] Zviedre, I. I. & Belsky, V. K. (2001). *Latv. Khim. Z.* **1**, 91–92.

